# Nanosilica Modification of Epoxy Matrix in Hybrid Basalt-Carbon FRP Bars—Impact on Microstructure and Mechanical Properties

**DOI:** 10.3390/ma16051912

**Published:** 2023-02-25

**Authors:** Karolina Ogrodowska, Marek Urbański

**Affiliations:** Faculty of Civil Engineering, Warsaw University of Technology, Al. Armii Ludowej 16, 00-637 Warsaw, Poland

**Keywords:** fiber, reinforced, polymer, bars, FRP, microstructure, hybridization, strength

## Abstract

This article focuses on the effect of nano-silica on an epoxy matrix of hybrid basalt-carbon fiber reinforced polymers (FRP) composites. Usage of this type of bar continues to grow in the construction industry. The corrosion resistance, strength parameters, and easy transport to the construction site are significant parameters compared to traditional reinforcement. The research for new and more efficient solutions resulted in the intensive development of FRP composites. In this paper, scanning electron microscopy (SEM) analysis of two types of bars is proposed: hybrid fiber-reinforced polymer (HFRP) and nanohybrid fiber-reinforced polymer (NHFRP). HFRP, in which 25% of the basalt fibers were replaced with carbon fibers, is more mechanically efficient than basalt fiber reinforced polymer composite (BFRP) alone. In HFRP, epoxy resin was additionally modified with a 3% SiO_2_ nanosilica admixture. Adding nanosilica to the polymer matrix can raise the glass transition temperature (Tg) and thus shift the limit beyond which the strength parameters of the composite deteriorate. SEM micrographs evaluate the surface of the modified resin and fiber–matrix interface. The analysis of the previously conducted tests—shear and tensile at elevated temperatures—correlate with the microstructural SEM observations with the obtained mechanical parameters. This is a summary of the impact of nanomodification on the microstructure–macrostructure of the FRP composite.

## 1. Introduction

The increasing importance of using high-performance materials in construction has led to a great interest in the development of fiber-reinforced composites in civil engineering. This type of composite is based on the reinforcing fibers which are coated in the matrix. Due to good bonding with reinforcing materials, low curing shrinkage, excellent dielectric properties, and resistance to chemical corrosion, epoxy resin is most often used as a matrix in composites used as elements of infrastructure structures. The FRP composite has properties of high tensile strength, resistance to the aggressive environment, easy transport and assembly, successfully replacing traditional steel reinforcement. To find composites with stable and high mechanical parameters, modifications related to fiber–hybridization [1,2,3,4] and the matrix–nano modification by SiO_2_ [5,6,7], other nanofillers [8,9,10,11,12], and additives [13] are used.

The experiment we carried out shows that modifying epoxy resin with nanosilica can increase parameters, such as the composite elastic modulus and glass transition temperature (Tg). This is very promising as these are the main parameters considered when designing structures, according to the American Concrete Institute (ACI). For example, increasing the modulus of elasticity for the footbridge structure by 10–15% has a beneficial effect on the load capacity. The increasing Tg value allows the strength parameters to be kept at the same level at higher operating temperatures and to consider value increases concerning the environmental reduction factor CE for FRP composite. Obtaining composite stability at high temperatures would allow the safe use of FRP composites in structures without significantly reducing their parameters during the design calculations. For performance and serviceability, concrete structures reinforced with FRP composites are very important, along with upgraded bonds of fiber-reinforced polymer bars with concrete. The nanomodification can grow the ultimate bond strengths, for example, between carbon FRP bars and concrete about ~37.8% [7,9].

### 1.1. Presentation of the State of the Art

The authors of the literature [14,15,16,17,18,19,20,21,22,23,24,25,26,27] conducted scientific studies on matrix modification. The 14 articles were analyzed. The size of the nanoparticles was in the range of 7–1050 nm. The research presented the influence of nanomodification on such properties as modulus of elasticity, tensile strength, and glass transition temperature.

In the analyzed papers (Table 1), parameters such as polymer binder, type of hardener, mixture homogenization method, and conditions of the resin curing process, i.e., time and temperature, were different. The results of the studies confirm the thesis that to obtain high-performance FRP composites, it is necessary to add the optimal amount of nanosilica, appropriate dimensions, and set the variables related to the binder production process at a constant level.

**Figure 1:**
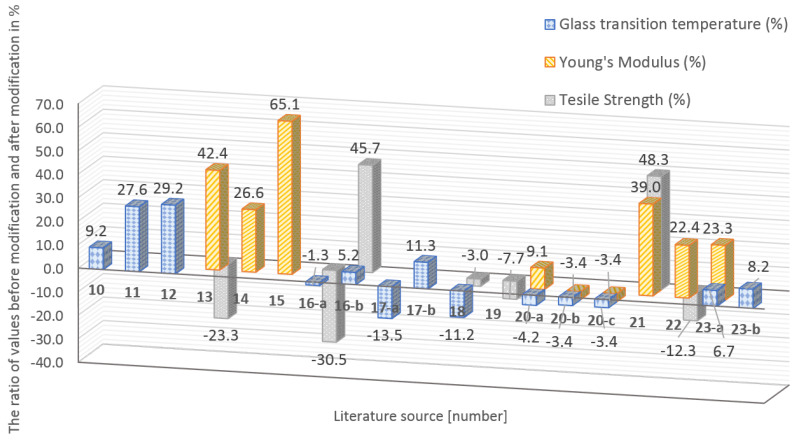
The graph presented the ratio of parameters before modification to parameters after modification of epoxy resin (Glass transition temperature, Modulus of elasticity, Tensile strength) expressed in %.

The compendium of analysis literature research [14,15,16,17,18,19,20,21,22,23,24,25,26,27]—impact on matrices properties:

- Modulus of elasticity. The papers [17,18,19,24,25,26,27] show the results of the nanomodification of various types of epoxy resin with nanosilica in sizes from 12 to 900 nm. All of the evaluated papers proved a positive effect of nanomodification on the modulus of elasticity (Figure 1);
- Tensile strength. In the papers [17,20,22,23,25,26], the influence of nanomodifications on the tensile strength of the matrix was investigated. The X > 10% change in original tensile strength was considered significant. In the papers [17,20,26], a significant decrease in tensile strength was found, respectively, by 30.5%, 23.3%, and 12.3%. On the other hand, works [20,25] showed a significant increase in tensile strength by 45.7% and 48.3% (Figure 1);
- Glass transition temperature. The influence of nanosilica on the Tg of the tested matrices was also analyzed. The ratio value of Tg after the addition of SiO_2_ to Tg before nanomodification was >1 for cases [14,15,16,20,21,22,24,27]. The positive effect of SiO_2_ nanomodification on Tg has been observed (Figure 1).

### 1.2. Purpose of the Work

The issue of FRP composites and their modification is more well-known in the use of FRP composite as a laminate, especially in the automotive industry, equipment lateral body production, aircraft machine manufacturing industries, structural and electronic elements, and the domestic sector. In civil engineering, a composite in the form of a bar is a fully innovative material. The search for increased strength and thermal parameters must also meet economic requirements so that the use of innovative composite bars in the construction sector competes with the use of traditional steel bars.

This paper deals with the hybrid basalt–carbon fiber reinforced polymers composite bars. The nanomodification of the composite matrix was applied. Next, we measured the influence of bar modifications on the most important mechanical parameters from the point of view of the constructor of civil engineering objects. The investigation of the previously conducted in elevated temperature tests is an indirect check of the influence of nanomodification on the Tg of the tested material. The FRP bar micrographs were recorded by SEM. The surface of modified and unmodified resin was determined quantitatively and qualitatively. The appropriate course of microstructural analysis was proposed for FRP composites usage in construction.

One of the principal elements in obtaining the strength parameters of a composite is interfacial strength [28,29,30,31,32,33]. To control the strength of the fiber-matrix bond of the bar, a shear test was used. According to Table 2, the interlaminar shear test has a very strong effect on matrix adhesive and the mechanical performance of the composite material. In the case of bars, the interlaminar shear is not as important as in the laminate/plate composites. The transverse shear test can assess whether the tested bar is sensitive to transverse force.

**Figure 2:**
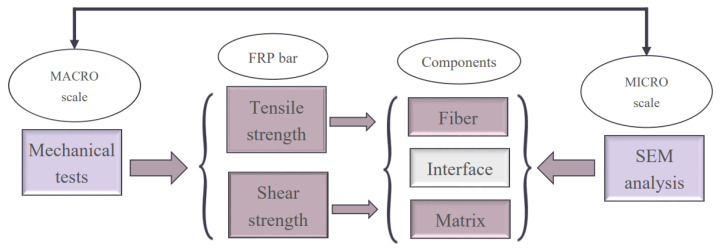
The author’s scheme of the macro–micro-scale research approach uses mechanical tests and SEM analysis. Micro-level models [1 μm] describe the structure and properties of the matrix/fiber; meso-level models [1 mm] record matrix-fiber interactions, including pores, inclusions, and microscopic scratches; macro-level models [1 dm] refer to matrix and fibers as a composite bar.

In our research, the scheme presented in Figure 2 was used. Examining the effect of nanomodification on tensile strength made it possible to assess the effect of nanomodification on a key parameter from the point of view of civil engineering and to compare the obtained data with the literature, the modulus of elasticity and tensile strength. This comprehensive combination of the evaluation of two tests and the SEM analysis allowed for analyzing the mechanisms occurring in the FRP composite under the applied load.

## 2. Material and Methods

### 2.1. Materials

The epoxy system (system name: “1300”), which was used in research bars, was composed of the following ingredients:

- Ingredient E—Epidian 1300, obtained from bisphenol A and epichlorohydrin;
- Ingredient H—Hardener 1300 anhydride type;
- Ingredient A—Accelerator 1300, applicable to accelerate the crosslinking process of epoxy resin;
- Ingredient M—Modifier 1300—polypropylene glycol diglycid ether. The modifier as an active diluent makes the resin more flexible.

**Figure 3:**
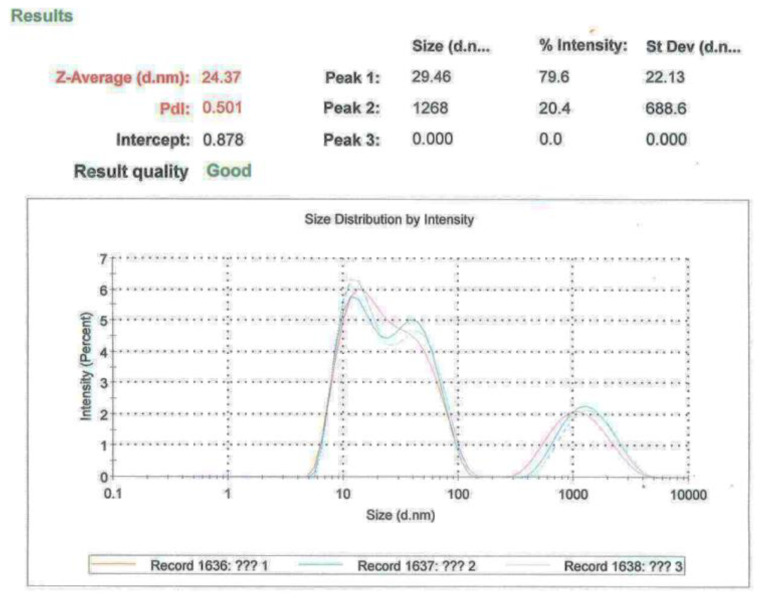
The particle size distribution of nanosilica used to modify the epoxy resin.

The combination “1300” was used to produce structural composites by pultrusion. All the chemical components of the matrix were supplied by Ciech Sarzyna S.A. company. Corresponding with the manufacturer’s instruction, the above ingredients were mechanically stirred. The components were distributed E:H:A:M = 100:70:5:7 (weight ratio). For the NHFRP bars, nanosilica was added to the matrix mixture. The amount of the nanosilica was measured by a Malvern apparatus. The average size of nano SiO_2_ used was 24.37 nm. There were two fractions. The finer with a peak at 30 nm—about 80%, and the coarse-grained with a peak at 1270 nm—about 20% (Figure 3). The unmodified bars were named HFRP, while the bars including nano SiO_2_ were named NFRP.

In this paper, nanosilica was used, which has been tested and subscribed to in the article [34]. P. Sikora nd others subject to nano SiO_2_ impact on the mechanical properties of polymer-cement composites (PCC). In this research, the SiO_2_ effect of 100 nm and 250 nm diameter, and 1%, 3%, and 5% quantity by weight of cement on the consistency and mechanical properties of PCC mortars, were investigated. Based on the tests carried out on the same type of nanosilica [34], the decision was made to introduce SiO_2_ in the amount of 3% into the composite matrix of the tested bars (Table 3).

Both types of bars—HFRP and NHFRP, were made by the manufacturer TohoTenax in the pultrusion process. The pultruded profiles have a high volume fraction of fibers of up to about 70%, and a constant product quality. Pultrusion is a highly automated process for manufacturing composite profiles from a fiber-reinforced polymer with a constant cross-section. When developing composite rods, it was assumed that the ratio of fiber to matrix volume would be 70:30. However, the actual fiber content (Table 3) was slightly modified due to the technological difficulties of the production process.

### 2.2. Methods

The number of samples for the transverse shear test were 20–10 NHFRP and 10 HFRP (Table 4). The shear test was carried out on the directive of ACI 440.3R-04 [35]. The test was prepared for the needs of the project. Details of the stand and study can be found in the works [36,37]. The samples for the tensile test were also 20–10 NHFRP and 10 HFRP. The tensile strength test was realized with the rules in the [35] standard for pultruded FRP bars. The details focused on the tensile test are in the paper [36,37].

The glass transition temperature provided by the manufacturer of the developed bar was 60 °C. The composites in the construction are in specific temperature phases related to the state of the resin. Stage I (20–100 °C)—insensitivity to elevation temperature. Stage II (200–400 °C)—oversensitive to temperature—when composite works at a temperature superior to Tg, the epoxide softens and melts.

**Figure 4:**
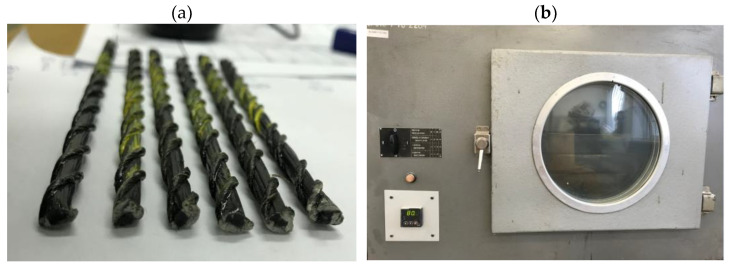
Preparation samples for mechanical tests; (**a**) One part of the samples prepared for the shear test, (**b**) The heating process of the samples at a stabilized temperature of 80 °C.

In this investigation, the temperature above Tg was taken at 80 °C, while the temperature of the second phase was assumed to be 200 °C. The heating time of 2 h results from the assumed full load capacity of the element for 240 min at elevated temperature. The samples were heated after the furnace stabilized to the set temperature (Figure 4b).

**Figure 5:**
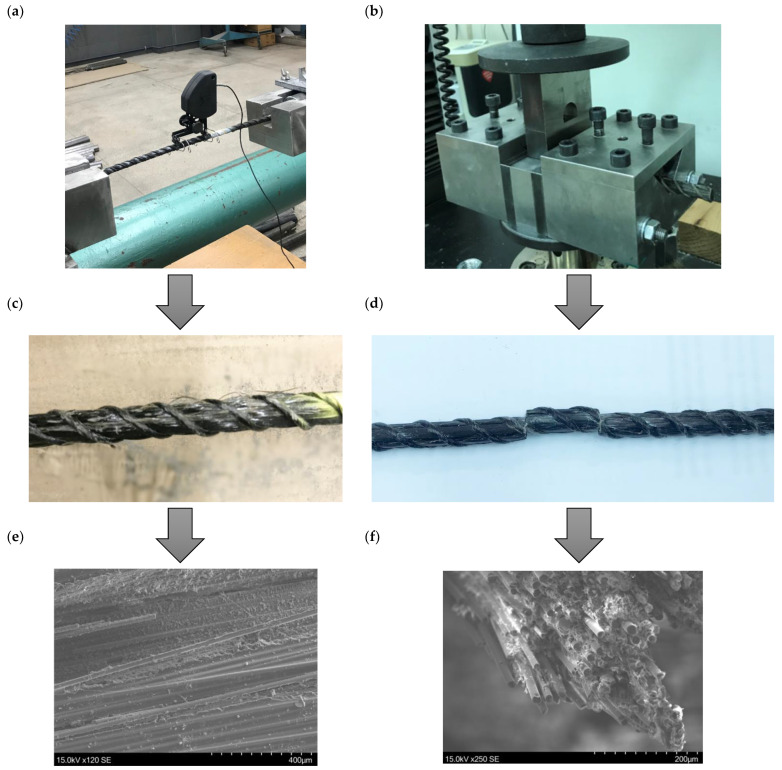
The previously performed test. (**a**) The tensile strength test—bar sample placed in a testing machine; (**b**) The transverse shear test—a bar sample placed in a shear apparatus; (**c**) The view of broken fibers after the tensile test; (**d**) The cut bar after the transverse shear test. The SEM micrographs of the bar after (**e**) tensile test; (**f**) shear test.

The developed bars were previously subjected to a shear (Figure 5b) and tensile test (Figure 5a) after heating for 2 h to 80 °C and 200 °C. A detailed description of the preparation of the samples and the performance of the strength tests can be found in the authors’ previous works [36,37].

After finalizing the strength tests (shear—Figure 5d, and tensile tests—Figure 5c), samples were taken for SEM tests (Figure 5e,f). The bars were cut transversely mid-length and about 2 cm from the middle. The exposed cross-sections were then polished with abrasive papers with a gradation of 800, 2000, and 2500. The specimens were washed, sputtered with a covering of gold 10 nm thick to obtain electrical conductivity, and placed on the microscope stage using carbon tape. The SEM photos (Figure 5e,f) of cross-sections were taken in two places (as described above, in the middle of the length and 2 cm from the middle) at 250× magnification, which allowed us to obtain a field of view of approximately 30 × 40 um. Analogous photos of the surface of the rods ("matrix") were also taken in similar conditions, with a lower magnification of 120×.

The SEM micrographs were taken with a Hitachi SU3500 scanning microscope using SE (secondary electron) and BSE (backscattered electron) detectors. The accelerating voltage was set to 15 kV, and the beam current to 60% (the current is not measured by the device, the "spot size" value is set in the range of 10–100%). The acquisition time for one image was 40 s.

## 3. Results and Discussion

### 3.1. SEM Analysis

The introduction of nanosilica into the mixture leads to an increase in viscosity. The uncontrolled increase in viscosity can lead to the formation of agglomerates. Papers researching the influence of nanosilica agglomeration on the behavior of cement-based materials are available [38,39]. In the paper [38], the nanoindentation test noted that low mechanical properties could be caused by weak zones created by large agglomerates.

**Figure 6:**
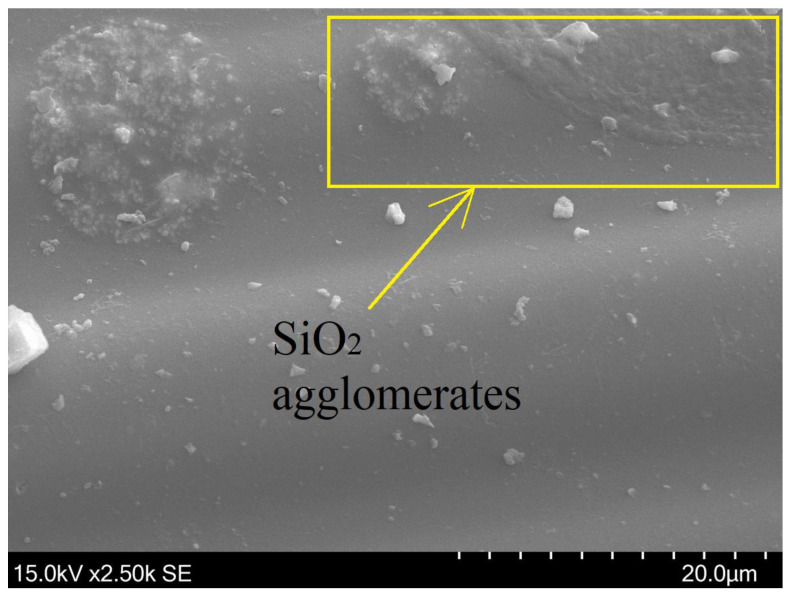
The surface of bar modified with nanosilica.

The agglomerated SiO_2_ particles (Figure 6) can negatively affect the bonding and are weak points in the composite structure. This could be one of the reasons for the decrease in the strength parameters of the tested composite. Adding an optimal amount of nanosilica avoids increasing the mixture’s viscosity and enables better processing.

The investigated fracture toughness tests and SEM observations in the literature paper [36] classified surface cracks and the toughening process. The epoxy matrices of FRP bars are breakable because of their highly crosslinked structure. This phenomenon relates to low resistance to crack initiation and propagation. The SEM images showed the exterior of the specimens. The fracture of pure epoxy resin was a smooth plane, indicating the fracture was brittle. The rough fracture surfaces were obtained after the epoxy was filled with nano SiO_2_. The more nanoparticles were inserted, the more plained the surface roughness [6,40]. This tendency was also noticed in fracture solidity [40].

**Figure 7:**
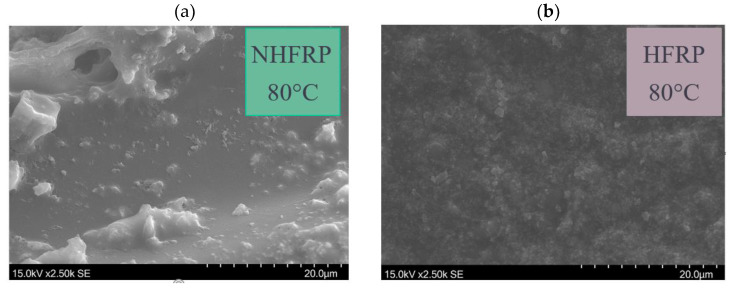
The surface of the matrix (**a**) NHFRP bar, which was pre-heated for 2 h at 80 °C; (**b**) HFRP bar, which was pre-heated for 2 h at 80 °C.

The same phenomenon was observed in this work. The rough surface of the matrix with the addition of nanosilica (Figure 7). It confirms the thesis that adding nanosilica increases the matrix’s resistance to brittle cracks.

**Figure 8:**
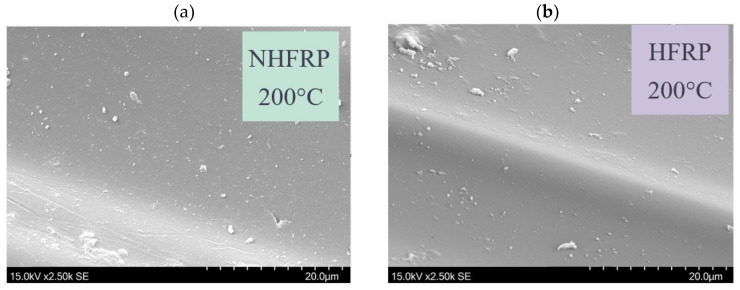
The surface of the matrix (**a**) NHFRP bar pre-heated for 2 h at 200 °C; (**b**) HFRP bar pre-heated for 2 h at 200 °C.

When the pre-heating temperature increased to 200 °C, it made the surface smoother (Figure 8). The epoxy matrix became more sensitive to brittle cracks. There was a weakening of the matrix–fiber interface. A negative effect of elevated temperature on the mechanical properties of the FRP composite was noted.

### 3.2. Tension and Shear Strength Tests

**Figure 9:**
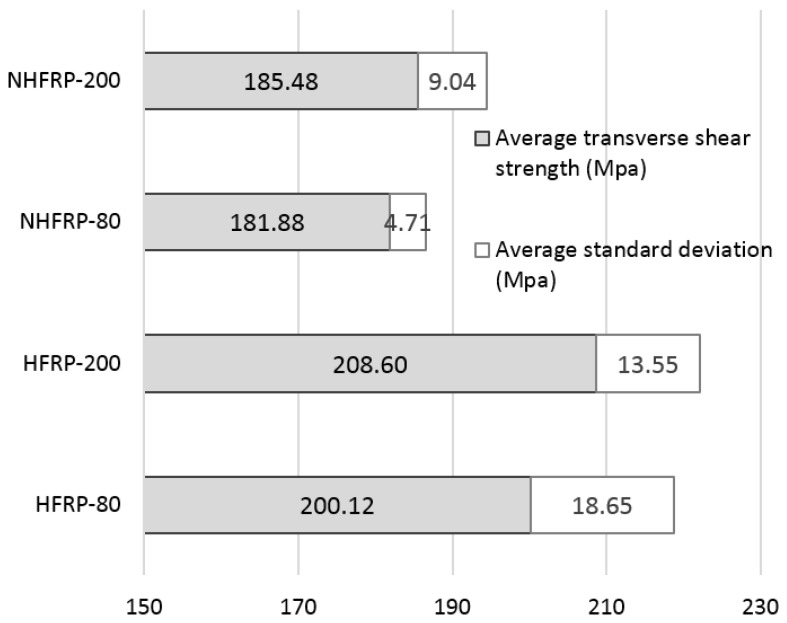
The average shear strength results for pre-heated bars to 80 °C and 200 °C.

The results of the transverse shear tests after pre-heating the samples are shown in Figure 9. The average shear strength for unmodified bars (HFRP) at 80 °C—200.12 MPa, 200 °C—208.06 MPa. The average shear strength for NHFRP composites is 80 °C—181.88 MPa, 200 °C—185.45 MPa. The nanomodification lowered the shear strength of the composite bars, while NHFRP bars have greater stability of the results at elevated temperatures. The increased shear strength after heating indicates insufficient curing time of the resin in the production process. After heating to 200 °C, the resin cured and increased its shear strength.

**Figure 10:**
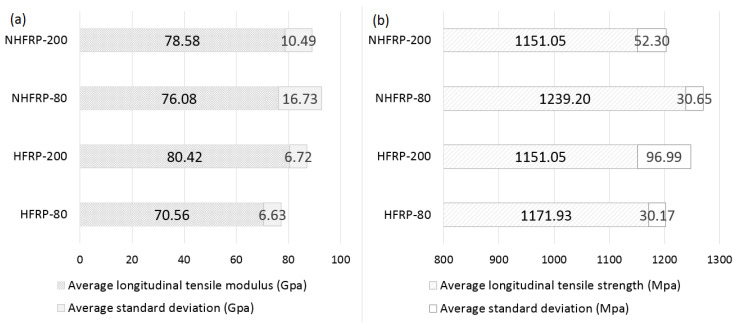
The average values for pre-heated bars to 80 °C and 200 °C: (**a**) tensile strength; (**b**) longitudinal tensile modulus.

The results of the longitudinal tensile tests after pre-heating the samples are shown in Figure 10a (tensile strength) and Figure 10b (longitudinal modulus of elasticity). The average tensile strength for unmodified bars (HFRP) at 80 °C—1171.93 MPa, 200 °C—1151.05 MPa. The average longitudinal tensile strength for modified bars (NHFRP) at 80 °C—1239.20 MPa, 200 °C—1151.05 MPa. The nanomodification increased the longitudinal tensile strength of the composite bars. After pre-heating to 200 °C, tested bars were observed to have a lower standard deviation than unmodified bars (Figure 10a).

In the case of Young’s modulus, similar results were observed for both HFRP and NHFRP bars at two temperatures. The average modulus of elasticity for unmodified bars ranged from 70.56 MPa to 80.42 MPa, while for modified bars, it oscillated around ~77 MPa. The standard deviation reached higher values for modified bars (Figure 10b).

In conclusion, agglomeration may have contributed to the formation of weak points in the composite space—the weakening of the transferred shear force after nanomodification. However, by increasing the surface roughness after the addition of SiO_2_, we obtained an increase in Young’s modulus due to the lower brittleness of the matrix, increasing flexibility and resistance to early crack propagation at a temperature of 80 °C.

## 4. Conclusions

The modification of composite matrix—epoxy resin with nanosilica is a very promising treatment to increase the performance of FRP’s bar composites. The proper introduction of SiO_2_ to the epoxy matrix should be followed to prevent the uncontrolled agglomeration of nanoparticles. The main effects observed by nanosilica modification of epoxy matrix are as follows:

(1) The results indicate that the addition of nanosilica improves the stability of the composite at elevated temperatures;
(2) Uncontrolled agglomeration of nanosilica particles can be the factor affecting weak points in the composite. The important factor is homogeneity. Use of additional treatments to obtain better homogeneity, e.g., the use of ultrasound. Additional research is needed;
(3) The SEM observations showed an increase in the matrix surface roughness at 80 °C. This had a positive effect on preventing crack propagation, especially in tensile strength;
(4) After exposure composite bar to a temperature of 200 °C, the surface smoothed;
(5) Stress-transferring boundary layer is weakened. The matrix plasticizes, as is shown in the study of the modulus of elasticity, which slightly increases at elevated temperatures.

The optimal production factors of FRP nanosilica-modified bars, such as homogenization and the resin curing method, can improve the "compatibility" of the composites and have a positive effect on the modulus of elasticity, tensile strength, and the matrix-fiber interface. It is necessary to verify the purpose of the nanomodification process, the share of changed parameterss and their impact on the structure reinforced with FRP bars.

## Figures and Tables

**Table 1:** The important parameters in the polymer processing affecting the final performance of the matrix [14,15,16,17,18,19,20,21,22,23,24,25,26,27].

No.1	Literature Source	Type of Polymer Binder	Type of Resin Hardener	Method of Homogenizing the Mixture	Hardening the Resin Process	
					Curing Temperatures	Total Time of the Process
1	[14]	Bisphenol F epoxide diglycidyl ether	EpiCure-W (dietylo-diamino-toluen)	Magnetic stirrer (3 h) + ultrasonication	room temperature, 121 °C–177 °C	5 h
2	[15]	Bisphenol A epoxide diglycidyl ether (DGEBA)	Polyamine amide adduct	Mechanical stirrer (1 h) + ultrasonication (1.5 h)	room temperature	5 days
3	[16]	Bisphenol A (DGEBA)	Methyl tetrahydrophthalic anhydride (MTHPA)	Mixing + ultrasonication	No data	No data
4	[17]	Bisphenol A with epichlorohydrin	Ethylimidazole	Mechanical stirrer (2 h)	140 °C	4 h
5	[18]	Epoxy resin	Liquid anhydride	No data	90 °C, 150 °C	4 h
6	[19]	Bisphenol F epoxide diglycidyl ether (DGEBF)	2-ethyl-4-methylimidazole and hexahydro-4-methylphthalic anhydride	Mixing (2 h)	60 °C, 150 °C	6 h
7	[19]	Cycloaliphatic resin	2-ethyl-4-methylimidazole and hexahydro-4-methylphthalic anhydride	Mixing (2 h)	125 °C, 200 °C	4 h
8	[20]	Epoxy resin	Modified cycloaliphatic amine	Mechanical stirrer (1 h) + ultrasonication	room temperature	7 days
9	[21]	Bisphenol A (DGEBA)	Cycloaliphatic polyamine	Mechanical stirrer + ultrasonication	room temperature	No data
10	[22]	Reaction product of bisphenol A	Ethylimidazole	Mechanical stirrer (2 h)	140 °C	4 h
11	[23]	Epoxy resin	Hardener based on amines	Mixing + ultrasonication (2 h)	40 °C	16 h
12	[24]	Epoxy resin (3,4-epoxycyclohexylmethyl-3,4-epoxycyclohexanecarboxylate—ECC)	Hexahydro-4-methylphthalic anhydride (HMPA)	No data	150 °C, 180 °C	11 h
13	[25]	Epoxy resin based on bisphenol A diglycidyl ether (DGEBA)	Piperidine	Mechanical stirrer	160 °C	6 h
14	[26]	Epoxy resin (DGEBA)	Piperidine	Mechanical stirrer	160 °C	6 h
15	[27]	Epoxy resin based on bisphenol A diglycidyl ether (DGEBA)	Poly (oxypropylene) diamine	Mechanical stirrer + ultrasonication	room temperature	48 h

**Table 2:** Effect of matrix adhesive on mechanical properties of composite material [26].

Composite Strength	Recommended Test Method	The Influenced Performance of Adhesive	Effect Sensitivity	Description
Flexural strength σ_Fu_	Three-point bending test	(1) Tensile modulus E_m_	Weak	Damage starts from tensile or compression surface
Longitudinal tensile strength σ_Lu_	Longitudinal tensile test Three-point bending test	(1) Tensile modulus E_m_ (2) Interface bond strength	Weak	For composite with high interlaminar shear strength, very sensitive to gap
Longitudinal compressive strength σ_Lu_	Longitudinal tensile test Three-point bending test	(1) Compressive strength (2) Tensile & shear modulus E_m_, G_m_ (3) Interface strength	Strong	Avoid whole buckling and cracking of the end; For low tensile modulus of the resin and the environment-sensitive resin, use bending samples
Interlaminar shear strength τ_u_	Shear test Short beam bending test	(1) Shear modulus (2) Ultimate strain (3) Interface strength	Very strong	It is very convenient for the evaluation of the material, process, and controlling performance

**Table 3:** Types of tested bars. The amount of the specific fiber fraction in the total fiber fraction and the composite’s mass.

Type of Bar	Basalt Fiber Content in the Fiber Fraction	Carbon Fiber Content in the Fiber Fraction	Fibers in the Composite Weight	Content SiO_2_ in the Matrix
HFRP	75.3%	24.7%	65.5%	-
NHFRP	75.3%	24.7%	65.6%	3%

**Table 4:** The specification of samples prepared for mechanical tests.

Type of Bar	Pre-Heating		Number of Samples to Mechanical Tests after Pre-Heating	
	Temperature	Time	Shear Test	Tensile Test
HFRP	80 °C	2 h	5	5
HFRP	200 °C	2 h	5	5
NHFRP	80 °C	2 h	5	5
NHFRP	200 °C	2 h	5	5

## Data Availability

The data presented in this study are available on request from the corresponding author.
